# Impedance changes in chronic vagus nerve stimulator implantation in epileptic dogs

**DOI:** 10.1007/s11259-026-11362-6

**Published:** 2026-06-18

**Authors:** Thomas R. Harcourt-Brown

**Affiliations:** Langford Vets, Lower Langford, Bristol, BS40 5DU United Kingdom

**Keywords:** Canine epilepsy, Vagus nerve stimulation, Impedance

## Abstract

Computational modelling of the electrical fields generated by helical electrodes surrounding the vagus nerve suggested a current of 1.0 mA should depolarise enough axons to give a therapeutic effect in epilepsy. An assumption that fibrous tissue formation would form between the electrodes and the nerve in implantable vagus nerve stimulators (VNS) led to the recommendation that 1.5 mA should be used to account for the increased impedance this would cause. This increase in impedance has not been documented, meaning that the higher current might not be necessary and could produce avoidable adverse effects such as coughing. To test the hypotheses that impedance does not always increase following VNS implantation and that more adverse effects might be seen with lower impedance circuits, circuit impedance was measured in dogs with implantable VNS at implantation and compared to impedance at 3–6 months post-implantation and at non-standardised time points thereafter. Dogs were grouped by severity of their coughing and the impedance between groups at 3–6 months was compared. Mean impedance at first implantation (2219Ω, 95% CI 1894–2544) was no different from impedance at 3–6 months post implantation (2257Ω, 95%CI 1944–2570, *p* = .80). Only 2 of 17 dogs showed a > 50% increase in impedance (one at 3–6 and one at 7–12 months). Coughing severity was not influenced by impedance value despite similar stimulation currents. This work shows impedance does not consistently increase post implantation in dogs with VNS, suggesting that currents > 1.0 mA might not be routinely needed to achieve a therapeutic effect.

**Supplementary Information:**

The online version contains supplementary material available at https://doi.org/10.1007/s11259-026-11362-6.

## Introduction

Implantable vagus nerve stimulation devices use coil electrodes to deliver a fixed current to a portion of the vagus nerve. This generates an electrical field proportional to the potential difference within the endoneurium between the electrodes.

Computational modelling has been used to calculate the electrical field strength to depolarise > 50% of the B-fibers within the vagus nerve as these are thought to be responsible for the therapeutic effect of VNS in the treatment of epilepsy (Helmers et al. 2012). An assumption made during this modelling was that fibrous tissue is likely to form between the electrodes and the nerve in the first 8 weeks after implantation. This was assumed to increase the circuit’s resistance sufficiently that higher currents would be required after 8 weeks post implantation to generate the same field strength as before fibrous tissue formed at implantation. This led to the recommendation that 1.5 mA current was required in patients after 8 weeks post implantation despite 1 mA being thought to depolarise sufficient axons if fibrous tissue was not present.

The most common adverse effect of VNS in dogs is coughing (Muñana et al. 2002; Harcourt-Brown and Carter 2021). The likelihood of coughing appears to increase in proportion to the current delivered (Harcourt-Brown and Carter 2021), so using the lowest current possible for therapeutic effect is desirable. Some dogs do not cough with currents that others cannot tolerate (Harcourt-Brown and Carter 2021). The reason for this is unclear, but it might be because the same current produces different electrical field strengths in different dogs because of variability in impedance and therefore different proportions of the axons responsible for coughing are activated during stimulation.

VNS circuit Impedance has not been shown to increase post implantation in the small number of dogs or humans for whom these data have been published (Martlé et al. 2016; Harcourt-Brown and Carter 2021; Cukiert et al. 2023), suggesting that the assumption of fibrous tissue forming between the electrodes and the nerve post implantation should be questioned. Additionally, the relationship between circuit impedance and coughing has not been investigated.

The aims of this study were to analyse the short- and long-term impedance changes in dogs with implantable VNS and document if dogs with severe coughing post implantation had lower impedance than dogs with less severe coughing.

## Materials and methods

The medical records of dogs with implantable VNS used to treat idiopathic epilepsy at a single institution between 2016 and 2023 were reviewed. Dogs were included if the impedance was measured at implantation and at least 1 time point more than 12-weeks post implantation. When multiple measurement were taken, they were grouped into the time periods 3–6 months, 9–12 months, 18–24 month, 30–36 months, and 44–48 months post implantation. If more than 1 measurement was taken during each 3-month period, only the last measurement was recorded.

The severity of coughing was recorded from the clinical notes at the same visit where data for the 3-6-month impedance value was obtained. This was graded using these definitions:

- None – no coughing observed or heard.
- Mild – coughs during stimulation a few times per day (rarely).
- Moderate – coughs during stimulation several times a day (often).
- Severe – coughs harshly or retches on most stimulations. This was considered intolerable for the dog and required alteration of VNS settings.

To analyse the short-term overall change in impedance, the impedance at implantation and the impedance 3–6 post implantation were compared using a paired, 2-tailed t-test after assessing for normality.

A 50% increase in impedance was considered to be clinically relevant. This figure was based on the assumption that most of the impedance recorded was caused by the tissue between the electrodes and the endoneurium (Grill and Mortimer 1994; Horn et al. 2023) so the impedance would be inversely proportional to the radial electrical field strength between the electrodes within the nerve, meaning a 50% increase in impedance would equate to a 50% reduction in field strength; the equivalent of a 50% reduction in applied current.

A post-hoc power calculation was performed (G*Power version 3.1.9.7, https://www.psychologie.hhu.de/arbeitsgruppen/allgemeine-psychologie-und-arbeitspsychologie/gpower) to estimate the likelihood of detecting a 50% increase in the mean impedance 3–6 months post implantation (assuming the standard deviation remained the same as at implantation).

To analyse longer-term changes, impedance at implantation and 3–6 months, 9–12 months, 18–24 month, 30–36 months, and 44–48 months post implantation were compared using a mixed model analysis with an assumption of sphericity and a post-hoc test for trend in increasing or decreasing values over time. A limit of *p*<.05 was set for statistical significance.

To analyse impedance changes at an individual-dog level, the proportion with a > 50% change in impedance at 3–6 months or any later time point was calculated.

To evaluate the effect of impedance on coughing at 3–6 months, a Kruskal-Wallis test was used to compare median impedance among the 4 grades of coughing severity.

## Results

Data for 24 dogs were reviewed. 3 dogs were excluded because the generator model implanted could not report a value of impedance. 4 more were excluded because they did not have impedance data recorded after initial implantation due to euthanasia (*n* = 1 at 48 days), lead breakage (*n* = 1 at 36 days) or they did not return for follow up monitoring (*n* = 2). This meant data from 17 dogs were available for analysis. Impedance data from 9 of these 17 dogs at initial implantation and 3–6 months post implantation has previously been reported(Harcourt-Brown and Carter 2021).

Four of these 17 had their leads replaced at 3, 9, 15 and 19 months after implantation. All were suspected to have lead damage caused by abrupt cessation in coughing. When checked, 3 had impedances > 10,000Ω and 1 was < 600Ω. 2 had their leads were replaced and 2 had their lead and generators replaced. These values at the time of failure or after re-implantation were not included in this analysis.

**Fig. 1:**
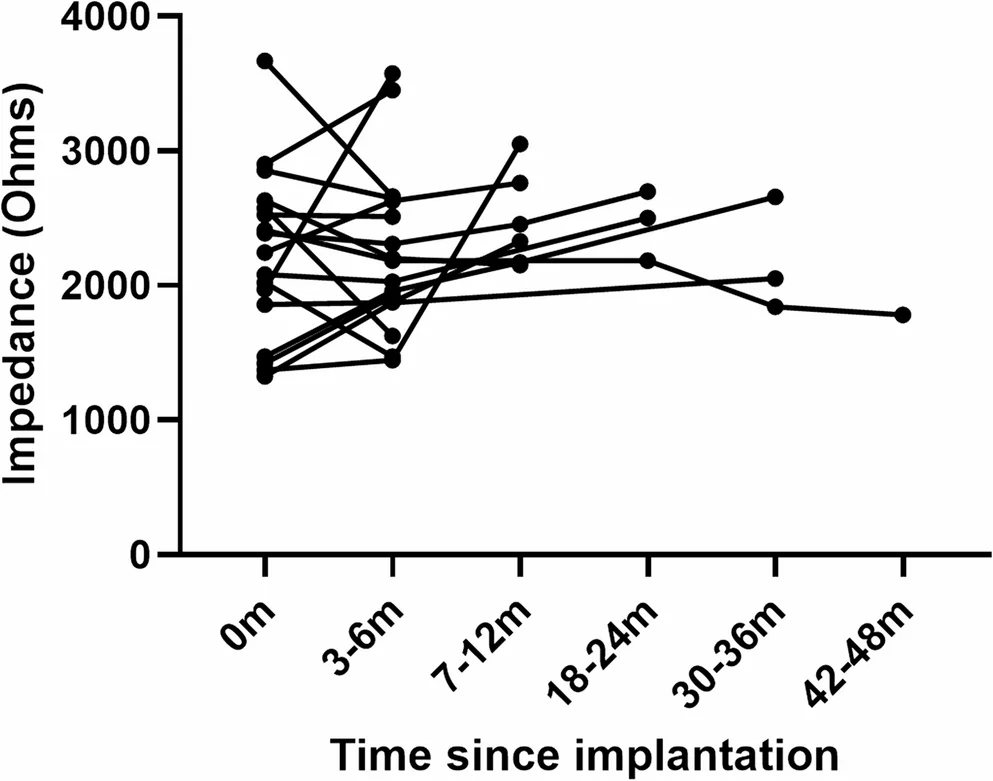
Impedance changes over time for all dogs included in the study

Mean impedance at first implantation (2219Ω, 95% CI 1894–2544) was no different from impedance at 3–6 months post implantation (2257Ω, 95%CI 1944–2570, *p* = .80, Fig. 1). Post-hoc power calculation estimated a power (1-β) > 0.99 for an effect size of 1.27 using 17 dogs. This can be interpreted to mean that we had a greater than 99% chance of detecting a 50% change in mean impedance between implantation and 3–6 months.

Impedance values were available for 7 dogs at 7–12 months, 5 dogs at 18–24 months, 4 dogs at 30–36 months and 4 dogs at 42–48 months. Not all dogs contributed to each period so data > 7 months was available for 13 dogs with 4 last followed up to 12 months, 3 last followed up to 24 months, 2 last followed up to 36 months and 4 last followed up to 48 months (Fig. 1). Mixed-effects analysis did not show a significant effect of time on impedance (*p=.5716*).

One dog had > 50% increase in impedance at 3–6 m months and another had a subsequent > 50% increase at 7–12 months post implantation.

**Fig. 2:**
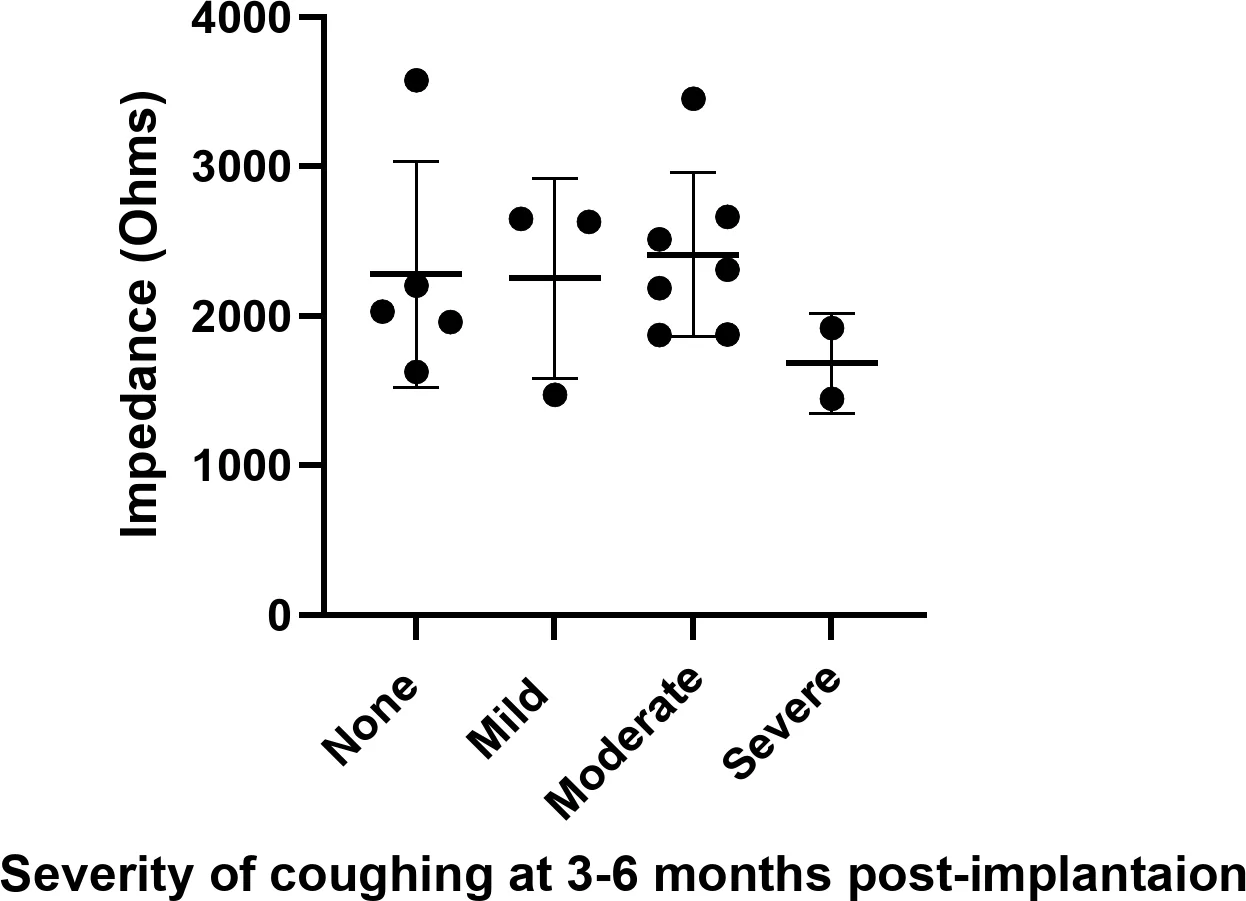
Impedance at 3–6 months for dogs grouped by severity of coughing at the time of measurement. Horizontal lines indicate means and one standard deviation

Median impedance at 3–6 months in dogs with no coughing (2277Ω, range 1624–3576Ω, *n* = 5), mild coughing (2629Ω, range 1473–2648Ω, *n* = 3), moderate coughing (2308Ω, range 1872–3543Ω, *n* = 7) or severe coughing (1682Ω, range 1446–1917, *n* = 2) was not different (*p=.3744*, Fig. 2).

## Discussion

We did not find a significant change in VNS circuit impedance in the first 3–6 months post implantation, or in the months to years post implantation in the dogs where data were available. Our findings are corroborated by recently published impedance measurements over time in epileptic children (Cukiert et al. 2023), where VNS impedance was found to decrease over time. We did not identify this decrease, perhaps because of species differences or the small sample size in both papers.

This stable impedance suggests that fibrous tissue is not forming between the electrode and vagus nerve sheath to a substantial degree in all dogs, so 1.0 mA (rather than 1.5 mA) may be sufficient to depolarise > 50% of the B-fibers within the vagus nerve(Helmers et al. 2012). This is an important finding as currents greater than 1 mA appear to cause more coughing in dogs during initial programming (Harcourt-Brown and Carter 2021), so aiming for a current of 1 mA rather than 1.5 mA could improve tolerability.

Human recommendations suggest that 1.0 mA should be the initial target for programming, but that this current should be increased as tolerability dictates, with an unreferenced statement that ‘some patients do not tolerate output currents greater than 1.0 mA (Heck et al. 2002). Translating our findings to human patients could indicate that increasing currents > 1.0 mA might not be necessary.

Computational modelling of axon depolarisation is not the only method for calculating optimal VNS settings. Clinical response has also been used, but although higher currents appear more effective than much lower currents (DeGiorgio et al. 2005) there does not appear to be a simple current-response relationship (DeGiorgio et al. 2001). A recent large retrospective analysis of the VNS manufacturers clinical records from 1178 human patients suggested there was a bell-shaped response centred around a current of 1.61 mA giving the greatest chance of > 50% seizure reduction at 12-months post implantation. No similar studies of dogs exist, but these data do suggest that the computational model of axon activation does not explain all the response to VNS and that further prospective studies of are needed to determine the optimum current for maximising seizure reduction.

We did not find that impedance differed among dogs with different severities of coughing. This suggests that coughing is not caused by simply activating more axons within the vagus nerve, otherwise, lower impedance between the electrodes and the nerve would activate more axons with the same current than circuits with a higher impedance. Instead, our finding is more consistent with the hypothesis that severity of coughing is determined by the proximity of the electrodes to the fascicles that become the recurrent laryngeal nerve. The closer these axons are to the electrodes, the more coughing will occur because the electrodes only cover around 270 degrees of the circumference of the nerve (Helmers et al. 2012). This hypothesis would require further testing with larger samples sizes to investigate its reliability.

Our study had several limitations. The first was the small number of cases included which will have reduced the impedance differences that we could detect. Our post-hoc power calculation for the number of dogs needed to detect a 50% increase in impedance over the first 6 months and the consistency of measurements in most dogs with longer follow-up suggest that we were unlikely to have missed a clinically relevant change over time at the group level. One of 17 dogs did have a > 50% increase in impedance within the first 6 months and another within the 7–12 month period suggesting that clinically relevant increases can occur in some dogs. Our small sample size makes it hard to accurately estimate how common this might be.

Another limitation is that we did not investigate the repeatability of impedance measurements. We took 1 measurement per check-up and there was a variable period between measurements. It is possible that multiple measures taken on the same day might give largely different results or that large fluctuations in impedance can occur in the weeks to months between measurements and we would not have detected that. We think this is unlikely because of the consistency of measurements we made over time in each dog, but it is an area where future investigations could be useful.

In conclusion, we found that impedance did not appear to predict coughing in the dogs we studied and that most dogs do not have significant changes in impedance over time, meaning 1.0 mA may be a suitable therapeutic current based on computational modelling. However, some dogs can have significant changes in impedance so this should be monitored at regular intervals and current increases in proportion to the impedance increase considered for those dogs based on their clinical response.

## Supplementary Information

Below is the link to the electronic supplementary material.

Supplementary Material 1

## Data Availability

All data supporting the findings of this study are available within the paper and its Supplementary Information. Impedance measurements used for analysis and the categorisation for coughing of each dog at 3-6 months are included in supplementary file A.
