# Supplementary material for: Impedance changes in chronic vagus nerve stimulator implantation in epileptic dogs
Source: Vet Res Commun. 2026 Jun 18;50(5):403. doi: 10.1007/s11259-026-11362-6 (PMC13279399; doi:10.1007/s11259-026-11362-6)
Supplement: Supplementary file 1 — Supplementary Material 1 [file 11259_2026_11362_MOESM1_ESM.docx]

| Dog | 0m | 3-6m | 7-12m | 18-24m | 30-36m | 42-48m | Cough at 3-6m |
| --- | --- | --- | --- | --- | --- | --- | --- |
| 1 | 2526 | 2511 |  |  |  |  | Mild |
| 2 | 1859 | 1875 | 2328 | *10000* |  |  | Mild |
| 3 | 2855 | 2648 |  |  |  |  | Mild |
| 4 | 1471 | 1959 | 2170 |  | 2658 |  | Moderate |
| 5 | 2020 | 1473 | *10000* |  |  |  | Moderate |
| 6 | 1970 | 3576 |  |  |  |  | Moderate |
| 7 | 1324 | 1872 |  |  | 2050 |  | Moderate |
| 8 | 1420 | 1917 |  |  |  |  | Moderate |
| 9 | 2630 | 2200 | 2150 |  |  |  | Moderate |
| 10 | 2900 | 3453 |  |  |  |  | Moderate |
| 11 | 2410 | 2186 |  | 2186 | 1843 | 1783 | None |
| 12 | 2083 | 2027 |  | 2503 |  |  | None |
| 13 | 2244 | 2629 | 2762 |  |  |  | None |
| 14 | 1371 | 1446 | 3053 |  |  |  | None |
| 15 | 2389 | 2308 | 2456 | 2697 |  |  | None |
| 16 | 3669 | 2661 |  | *10000* |  |  | Severe |
| 17 | 2574 | 1624 |  |  |  |  | Severe |

Impedance (ohms) measured at different time periods post VNS implantation. Measurements of 10000ohms represent device failure and are not included in analysis or figures
